# A Histone Methyltransferase Modulates Antigenic Variation in African Trypanosomes

**DOI:** 10.1371/journal.pbio.0060161

**Published:** 2008-07-01

**Authors:** Luisa M Figueiredo, Christian J Janzen, George A.M Cross

**Affiliations:** 1 Laboratory of Molecular Parasitology, the Rockefeller University, New York, New York, United States of America; 2 Department of Genetics, University of Munich, München, Germany; University of Oxford, United Kingdom

## Abstract

To evade the host immune system, several pathogens periodically change their cell-surface epitopes. In the African trypanosomes, antigenic variation is achieved by tightly regulating the expression of a multigene family encoding a large repertoire of variant surface glycoproteins (VSGs). Immune evasion relies on two important features: exposing a single type of VSG at the cell surface and periodically and very rapidly switching the expressed VSG. Transcriptional switching between resident telomeric VSG genes does not involve DNA rearrangements, and regulation is probably epigenetic. The histone methyltransferase DOT1B is a nonessential protein that trimethylates lysine 76 of histone H3 in Trypanosoma brucei. Here we report that transcriptionally silent telomeric *VSG*s become partially derepressed when *DOT1B* is deleted, whereas nontelomeric loci are unaffected. DOT1B also is involved in the kinetics of VSG switching: in ΔDOT1B cells, the transcriptional switch is so slow that cells expressing two VSGs persist for several weeks, indicating that monoallelic transcription is compromised. We conclude that DOT1B is required to maintain strict *VSG* silencing and to ensure rapid transcriptional *VSG* switching, demonstrating that epigenetics plays an important role in regulating antigenic variation in T. brucei.

## Author Summary

The surface of Trypanosoma brucei, a unicellular parasite that lives in the bloodstream of its mammalian host, is coated with glycoprotein (VSG) molecules. To evade elimination by the immune system, this parasite replaces its coat with one tailored from another glycoprotein variant. Even though there are hundreds of *VSG* genes in the genome, this process, called antigenic variation, works because all are silenced except for the one that encodes the current coat. In this work, we show that the chromatin-modifying enzyme DOT1B helps to epigenetically regulate the number of VSGs each parasite can have at a time at the surface and how fast each parasite can switch from one coat to another. In parasites lacking DOT1B, silent *VSG* genes become partially active and the switch from one VSG to another slows down, allowing two different VSGs to appear on the surface of an individual parasite at the same time. Our studies reveal the importance of epigenetics in regulating *VSG* genes and provide new insights toward the understanding of this unique survival device.

## Abstract 3

Antigenic variation in *Trypanosoma brucei* relies on monoallelic expression of a multigene family. New evidence shows that a chromatin-modifying enzyme prevents simultaneous expression of different proteins at the parasite's surface.

## Introduction

Post-transcriptional histone modifications play important roles in the regulation of chromatin structure and gene expression. Unlike acetylation, which is in general associated with transcription activation, histone methylation can activate or repress transcription depending upon the genomic location and the position of the modified amino acid in the histone chain [1]. Histone methylation mainly occurs on lysine or arginine residues that are located in the N-terminal tails of histones H3 and H4. One exception is lysine 79 of histone H3 (H3K79), which is located in the globular domain of H3 and is methylated by Dot1 in yeast [2,3] and hDOT1L in humans [4]. Very little is known about the function of H3K79 methylation. In yeast, it has a role in maintaining heterochromatin, probably indirectly, by limiting the spreading of Sir2 and Sir3 proteins into euchromatin [2]. In yeast and mammalian cells, H3K79 methylation appears to be involved in the detection of DNA damage [5,6] and in the development of leukemia as a result of *Hox* gene activation [7].

Antigenic variation is one of the most elegant systems that have evolved to evade host immune defenses. Trypanosoma brucei, the unicellular parasite that causes African sleeping sickness, expresses a single type of variant surface glycoprotein (VSG) at the cell surface and escapes the host immune response by periodically exchanging it with a different VSG (reviewed in [8]). The expressed *VSG* is always transcribed from one of the ∼15 bloodstream expression sites (BESs), which are always located at telomeres [9]. To ensure monoallelic *VSG* expression, only one BES is transcribed by RNA polymerase I at any time. This active BES localizes to a specialized extranucleolar compartment, the expression site body (ESB), which is proposed to contain the transcription machinery and regulatory factors that are required for complete processing of BES transcripts [10,11]. One of the mechanisms used to change the transcribed *VSG* is coordinated silencing and activation of different BESs. This stochastic process occurs at a low frequency but is very rapid. Attempts to select cells with two simultaneously active BESs revealed that switching intermediates are very unstable and short-lived [12]. BES switching does not seem to require DNA rearrangements [13], which suggests that it is mediated by epigenetic mechanisms. Although an ISWI homologue was shown recently to be involved in silencing BES promoter-proximal regions [14], no well-characterized chromatin remodeling factors are known to participate in *VSG* gene regulation.

The function and structure of chromatin in T. brucei is very poorly understood. DNA is not methylated, but it contains an unusual modified base β-glucosylhydroxymethyluracil (J) [15], which is mainly present in telomeric repeats and silent BESs [16] but whose function remains unknown. The histone tails of T. brucei are highly diverged from other well-studied eukaryotes. Nevertheless all core histones are subject to a few post-transcriptional modifications [17,18], including some unusual ones such as methylation of the N-terminal alanine residues of H2A, H2B, and H4. Accordingly, the genome of T. brucei contains candidates for multiple histone-modifying enzymes (reviewed in [19]), including two disruptor-of-telomeric silencing (DOT) methyltransferases, DOT1A and DOT1B, that are responsible for the methylation of H3K76 (corresponding to H3K79 in yeast and mammals). DOT1A is essential for viability and is responsible for dimethylation of H3K76, which is important for cell cycle regulation. DOT1B is not essential and exclusively trimethylates H3K76, a modification apparently required for complete differentiation into the insect stage of the life cycle in culture [20]. In this study, we examined the role of DOT1B in *VSG* transcriptional regulation. We found that DOT1B is required for complete BES silencing but not for regulating the expression of nontelomeric genes. When we selected for activation of a second BES in ΔDOT1B cells, we observed that many cells expressed two VSGs at their surface. With continuous selection, switching was eventually completed. We conclude that DOT1B is necessary for monoallelic *VSG* expression and rapid switching kinetics, which demonstrates the importance of a histone-modifying enzyme for *VSG* regulation.

## Results

### DOT1B Is Required for Complete BES Silencing

**Figure 1:**
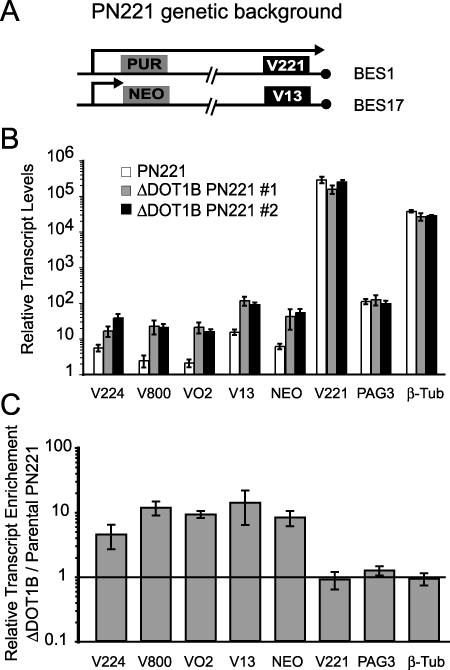
Disruption of DOT1B Results in Derepression of Silent *VSG*s (A) Schematic representation of PN221 genetic background, in which the actively transcribed BES1 is tagged with a *PUR* and the silent BES17 is tagged with *NEO*. *VSG221* (*MITat1.2*) and *VSG13* (*MITat1.13*) genes are located ∼50 kb downstream of the promoter (arrow) and ∼1 kb upstream of the telomere (circle). (B) Transcript quantification by quantitative RT-PCR of WT PN221 and two ΔDOT1B PN221 bloodstream clones, grown in the presence of 1 μg/ml puromycin. *VSG224* (*MITat1.3*), *VSG800* (*MITat1.18*), *VSG VO2* (*MITat1.9*), *VSG13*, and *NEO* are genes present in silent BESs, whereas *VSG221* is actively transcribed in BES1. *PAG3* is present in one procyclin locus, which is transcriptionally downregulated in bloodstream forms*.* β*-Tubulin* genes are present in a tandem array that is transcribed by RNA polymerase II. *Actin* was used as the reference gene. Error bars indicate the standard deviation of sextuplicates of the same cDNA sample. (C) Relative enrichment of ΔDOT1B transcripts relative to the wild-type parental reference (average of four independent measurements). When column height is >1, ΔDOT1B transcript levels are higher than those in parental PN221.

To test whether DOT1B is involved in transcriptional regulation of BESs, we generated a cell line in which genes conferring resistance to puromycin or G418 were introduced immediately downstream of the promoters of two BESs (Figure 1A), an approach first used in [21]. In a first step, the active BES1 was tagged with a puromycin resistance gene (*PUR*) downstream of the promoter. In silent BESs, transcription initiates at the BES promoter but is rapidly attenuated. This low level of transcriptional activity allowed the random integration of the neomycin resistance gene (*NEO*) immediately downstream of any “silent” BES promoter. PCR and pulsed-field gel electrophoresis genotyping showed that *NEO* had integrated at BES17. Because this double-tagged cell line actively expresses VSG221, we named it PN221. This reporter strain served two purposes: to eliminate cells that spontaneously silence either BES17 or BES1 and to place unique molecular markers into the otherwise highly conserved DNA sequences that flank BES promoters.

PN221 and two clones of ΔDOT1B PN221 were grown for at least 5 d in the presence of a high concentration of puromycin. This treatment killed any cells that may have undergone a BES switch, ensuring that the active BES in the entire population was BES1. Real-time quantitative reverse-transcriptase PCR (RT-PCR) was used to measure the transcript levels of several genes. In the absence of *DOT1B*, the transcripts of four silent *VSG*s and *NEO* were approximately 10-fold higher compared to those of the parental cell line, indicating that the absence of DOT1B reduces BES silencing (Figure 1B and 1C). The fact that both the promoter-proximal *NEO* and the telomeric *VSG13* of BES17 showed a similar increase in transcript levels suggests that the entire BES17 is partially derepressed. No significant differences were found for the highly expressed *VSG221*, whose transcripts are 10^4^–10^5^ more abundant than silent *VSG* mRNAs. No changes were seen in mRNA levels of non-BES genes, such as *actin* and β*-tubulin*, which are transcribed by RNA polymerase II, or for procyclin-associated gene 3 (*PAG3*), whose transcription by RNA polymerase I is largely repressed at this stage of the life cycle [22].

Taken together, these results show that DOT1B is necessary for complete BES silencing. Derepression due to loss of DOT1B was not observed in a non-BES gene, which suggests that the absence of this methyltransferase affects only a subset of genes, including the *VSGs*.

### DOT1B Is Required for Monoallelic *VSG* Expression

**Figure 2:**
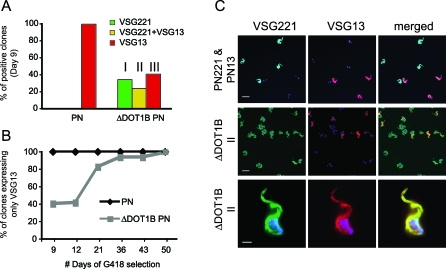
DOT1B is Required for Rapid Switching and for Monoallelic Expression (A) VSG expression of G418-resistant clones after 9 d of drug selection. Ten parental PN and 17 ΔDOT1B PN clones were analyzed by protein dot blot with anti-VSG221 and anti-VSG13 purified sera. The same analysis was repeated in three independent experiments, on an average of 15 G418-resistant clones. (B) Variation of VSG expression with time. The same clones described above were periodically analyzed by dot blot for a total of 50 d. The percentage of clones that are exclusively recognized by anti-VSG13 is plotted. (C) Non-cross-reactivity of anti-VSG221 and anti-VSG13 were tested by IFA on a 50:50 mixture of wild-type PN221 and PN13 cells (upper panels). The same sera and conditions were used for IFA of ΔDOT1B Class II clones (middle and bottom panels). DNA was detected with DAPI (blue). Scale bars: 10 μm (upper and medium panels), 2 μm (lower panel).

One of the mechanisms to change the VSG coat relies on transcriptional silencing of one BES and activating another. As the deletion of DOT1B affected *VSG* silencing, we hypothesized that BES switching might be affected by the absence of DOT1B*.* In vitro VSG switching occurs at a very low frequency, which means that the proportion of switchers of a population is so small that most methods cannot detect them. To overcome this problem, positive drug selection was used to select for cells that stochastically activated BES17. In the original PN221 reporter strain, the vast majority of cells are puromycin-resistant and G418-sensitive (unpublished data). When high concentrations of G418 (75 μg/ml) were added to 2 × 10^6^ cells of 5 to 12 PN221 parental or ΔDOT1B subclones, most cells died. Approximately 8 G418-resistant clones were obtained per subclone after 1–2 wk of incubation, which suggests that parental and ΔDOT1B clones switch at a similar frequency. Nine days after adding G418, protein dot-blot analysis showed that all G418-resistant clones from parental cells, but only a fraction of ΔDOT1B clones, expressed VSG13 (Figure 2A). Three classes of ΔDOT1B clones were detected (Figure 2A): those that exclusively expressed VSG221 (Class I), those that expressed VSG221 and VSG13 (Class II), and those that exclusively expressed VSG13 (Class III). With continuous G418 selection, VSG221-expressing clones became VSG221/VSG13 double-expressing clones, and these eventually expressed only VSG13 (Figure 2B). These results indicated that ΔDOT1B cells are capable of switching from BES1 (*VSG221*) to BES17 (*VSG13*) at a similar frequency as wild-type cells, but the switchover proceeds more slowly.

To study VSG expression in individual cells, indirect immunofluorescence analysis (IFA) was performed using purified anti-VSG221 and anti-VSG13. IFA on a mixture of PN221 and PN13 confirmed that both anti-VSG sera are specific (Figure 2C, upper panel). All cells in ΔDOT1B Class II expressed VSG221, but some also expressed VSG13 (Figure 2C, middle and lower panels), indicating that the VSG coat is composed of more than one type of VSG. Both VSGs seem to be uniformly distributed over the cell surface. The number of detectable double-expressers varied with time and between clones, ranging from 10–60% of the total number of cells. From these results we conclude that DOT1B is required for monoallelic *VSG* expression.

The presence of two VSGs at the surface of a cell suggested that two BESs might be simultaneously active. Because both BESs were tagged with selectable markers, we could use high-level resistance to puromycin or G418 as a measure of the transcriptional status of BES1 or BES17, respectively. Before selection with G418, VSG221-expresssing parental and ΔDOT1B cells were puromycin-resistant and G418-sensitive, which indicated that BES1 was active and BES17 silent. After G418 selection, switched parental cells were sensitive to puromycin, but ΔDOT1B Class I and ΔDOT1B Class II cells were resistant. In fact, both classes of ΔDOT1B cells were able to grow in the presence of both puromycin and G418 with a doubling time (∼7 h) only slightly longer than that of wild-type cells (Figure S1). In contrast, Class III cells were puromycin-sensitive. Pulsed-field gel electrophoresis and PCR analysis showed that no DNA rearrangements had occurred (unpublished data).

Taken together, these results (summarized in Table 1) show that DOT1B is necessary to maintain monoallelic expression in the cell population. In the absence of DOT1B, BES switching can still occur, but more slowly. A stable intermediate stage can be detected in which BES1 and BES17 are simultaneously active, conferring resistance to both drugs and exposing two VSGs at the surface.

**Table 1:**
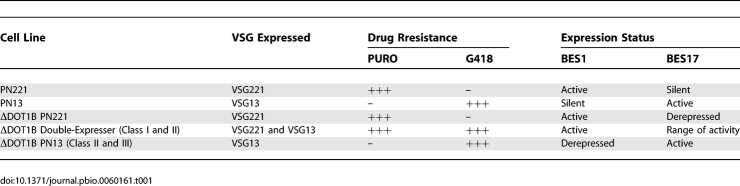
Phenotype of Wild-Type and ΔDOT1B Double-Tagged Clones

### DOT1B Is Required for Rapid Changes in BES Expression

**Figure 3:**
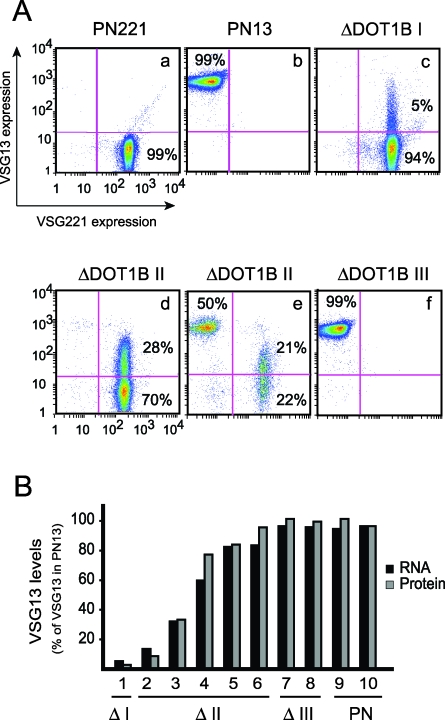
Two BESs Are Simultaneously Active in ΔDOT1B Double-Expressers (A) Living cells were stained with Alexa Fluor 488 anti-VSG221 and Alexa Fluor 647 anti-VSG13 and analyzed by FACS. Intensities of VSG221 and VSG13 staining are shown on the *x* and *y* axes, respectively. Percentages refer to the proportion of the total cells in a given quadrant. Each panel represents an independent clone: parental PN221 and PN13 clones (panels a and b), one ΔDOT1B Class I clone (panel c), two ΔDOT1B Class II clones (panels d and e), and one ΔDOT1B Class III clone (panel f). (B) *VSG* RNA and protein levels in multiple ΔDOT1B clones were measured by northern and FACS analysis and are indicated as a percentage of the levels in wild-type PN13 (sample #10). Five independent ΔDOT1B Class II clones expressing different levels of VSG13 were selected based on the quantification by FACS analysis. Pearson correlation between RNA and protein series is +0.99.

Closer analysis of IFA images revealed that ΔDOT1B Class II cells stained uniformly with anti-VSG221, but the intensity of anti-VSG13 staining varied widely from cell to cell. Flow cytometry was used to quantify cell-surface VSG. Parental cells exclusively expressing VSG221 (PN221) stained intensely with anti-VSG221 but not detectably with anti-VSG13 (Figure 3A, panel a). As expected, the reverse was true for parental cells expressing VSG13 (PN13) (Figure 3A, panel b). These results show that both antibodies are specific and can be used to analyze surface VSG levels in a mixed population of living cells.

The fluorescent-activated cell sorting (FACS) profile of ΔDOT1B Class I (Figure 3A, panel c) was very similar to that of PN221 cells, except that 5% of the cells were slightly VSG13-positive. The average intensity of this staining was very low (∼20 units), which probably explains why dot-blot analysis of Class I clones failed to detect VSG13. In two independent ΔDOT1B Class II clones (Figure 3A, panels d and e), the proportion of cells with detectable VSG13 was larger (28% and 21%) and detectable by dot-blot analysis (unpublished data). As observed by IFA, the VSG221 staining intensity of double-stained cells was constant and identical to that of parental PN221 cells, whereas VSG13 intensity varied between cells (Figure 3A, panel d). Interestingly, among ΔDOT1B double-expressers, the VSG13 intensity never reached the average intensity of PN13 cells (∼800 units), suggesting that the levels of surface VSG13 in double-expressing cells are below wild-type levels. After a variable number of days of G418 selection, a small distinct population appeared with VSG13 intensity as high as single VSG13-expressing PN13 cells and no detectable VSG221 (Figure 3A, panel e). This population of VSG13 single-expressers invariably became increasingly prominent over the double-expressers until it comprised the entire population. At this point, the VSG expression profile of these clones was identical to that of Class III clones (Figure 3A, panel f), which are undistinguishable from that of PN13 single-expresser (Figure 3A, panel b). These results show that, albeit slowly, ΔDOT1B cells eventually reach a VSG profile identical to that of a wild-type VSG13-expressing clone. It is noteworthy that, in the different classes of ΔDOT1B G418-resistant cells, we never detected a population with intermediate levels of VSG221, suggesting that BES1 is fully transcribed until it is rapidly and completely silenced.

To confirm that the levels of surface VSG and mRNA are correlated, RNA and protein levels in independent ΔDOT1B clones with variable levels of VSG13 expression were quantified by northern blotting and FACS analysis (Figure 3B). The correlation between *VSG13* mRNA and surface protein levels was very high (+0.99), indicating that surface VSG staining reflects steady-state mRNA levels and suggests that the ΔDOT1B switching intermediates simultaneously transcribe two BESs.

### Partial BES Activation Is Stably Inherited

**Figure 4:**
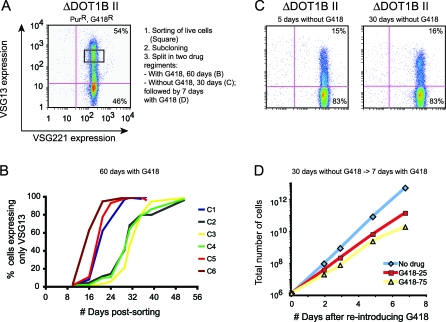
ΔDOT1B Double-Expressers Are Stable Switching Intermediates (A) A population of ΔDOT1B Class II cells brightly stained with anti-VSG221 and anti-VSG13 (square) was sorted and subcloned by limiting dilution either in the presence of 100 μg/ml G418 (60 d) or in the absence of any drug (30 d). (B) Six clones grown in the presence of G418 were submitted to FACS analysis every 8 d to follow the proportion of cells that exclusively express VSG13. (C) After 5 and 30 d without any antibiotics, VSG221 and VSG13 expression was measured by FACS. The profile of one representative clone is shown. (D) After 30 d without antibiotic, cells were diluted in 25 or 75 μg/ml G418, and cell growth was followed for 7 d.

After prolonged G418 selection, a population of cells exclusively expressing VSG13 appears among ΔDOT1B Class II clones, and this population eventually outgrows the double-expressing cells. To confirm that these pure VSG13 expressers originate from a double-expressing cell, FACS was used to sort only those cells that were brightly stained with both anti-VSG221 and anti-VSG13 (marked with a square in Figure 4A). These cells were subcloned and cultivated for 60 d in a high concentration of G418. FACS analysis showed that, in all subclones, a population of pure VSG13-expressers emerged between 8 and 24 d and gradually became more abundant, eventually leading to a homogeneous population of VSG13 expressers (Figure 4B). These results show that a ΔDOT1B double-expressing cell is capable of completing the switching process between BES1 and BES17, which confirms that a double-expresser is a switching intermediate.

To test whether partial activation of BES17 was dependent on drug selection, double-expressers were sorted, subcloned, and cultured without antibiotics for 30 d. Except for a larger proportion of cells with undetectable levels of VSG13 between 5 and 30 d postsorting, the FACS profile of the sorted cells (Figure 4C) was very similar to the profile of the starting population (Figure 4A). When a high concentration of G418 was added back to the medium, no cell death or growth lag was observed, and cells continued to grow exponentially, only slightly slower than in the absence of drug (Figure 4D). These results showed that partial activation of BES17 persisted and was stably inherited in the absence of drug pressure.

### Other VSG Genes Remain 10-Fold Derepressed in Double Expressers

**Figure 5:**
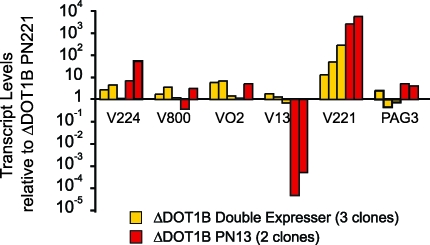
Silent *VSG*s Remain 10-Fold Derepressed in ΔDOT1B Double-Expressers Transcript levels of three independent ΔDOT1B double-expresser clones expressing different levels of VSG13 were compared to one ΔDOT1B PN221 and two ΔDOT1B PN13 single-expressers, by quantitative RT-PCR. *VSGs 224*, *800*, and *VO2* are present in silent BESs. *VSG221* is maximally transcribed in ΔDOT1B PN221 and ΔDOT1B double-expresser clones and silent in ΔDOT1B PN13. VSG13 is present in BES17, which is partially active at different levels in the three ΔDOT1B double-expressers and fully active in ΔDOT1B PN13. *PAG3* is present in one procyclin locus, which is transcriptionally downregulated in bloodstream forms*.* β*-tubulin* was used as the reference gene. Transcripts levels are indicated relative to ΔDOT1B PN221.

If DOT1B only affects the kinetics of specific drug-selected BES switching, then we expected *VSG* genes in other BES to remain repressed in ΔDOT1B double-expressers. Transcript levels of silent telomeric *VSG*s 224, 800, and VO2 were measured by quantitative RT-PCR. Three ΔDOT1B double-expressing clones expressing different levels of VSG13 were compared to ΔDOT1B single-expressers (one ΔDOT1B PN221 and two ΔDOT1B PN13 clones). Figure 5 shows that transcript levels of silent *VSG*s in the three ΔDOT1B double-expressing clones were not significantly different from the levels in the single-VSG-expressing clones ΔDOT1B PN221 and ΔDOT1B PN13. The control gene, *PAG3*, also remained essentially unchanged. VSG221 transcript levels in ΔDOT1B double-expressers were identical to those of ΔDOT1B PN221, in agreement with the FACS data, which showed that BES1 remained fully active in these two types of clones (Figure 3A). In contrast, in ΔDOT1B PN13, VSG221 transcripts were, on average, 3 × 10^4^-fold lower, which reflects the rapid silencing of BES1. These results confirm that silent *VSGs* remain largely repressed in ΔDOT1B double-expressers, displaying only the basal 10-fold derepression described previously for ΔDOT1B PN221 expressers (Figure 1).

### Partially Active BES17 Is Not Found in a New Extranucleolar Body

**Figure 6:**
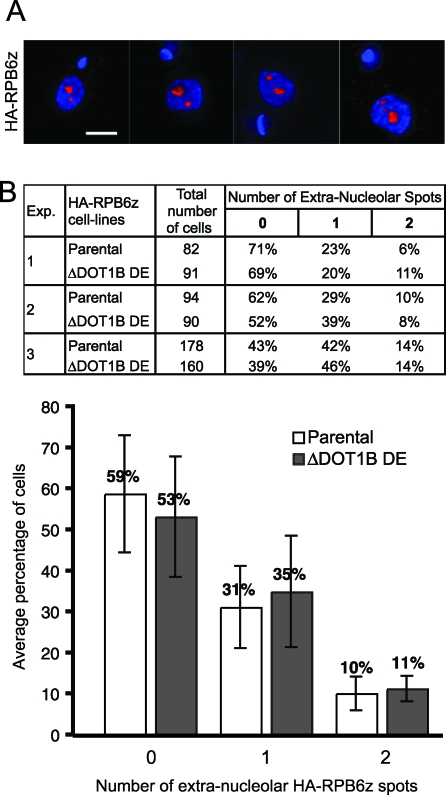
Frequency of Extranucleolar Spots Is Identical in Parental and ΔDOT1B Double-Expresser HA-RPB6z Clones (A) Examples of four nondividing bloodstream cells expressing HA-RPB6z, detected by HA.11 monoclonal antibody. DNA was detected with DAPI (blue). Scale bar: 2 μm. (B) Quantification of cells containing 0, 1, or 2 extranucleolar spots in parental and ΔDOT1B double-expresser (DE) HA-RPB6z clones. The total number of cells and the percentage in each category is indicated for three independent experiments. The average values from the three experiments are shown as a histogram.

The active BES localizes in the extranucleolar ESB [10]. It has been proposed that limited access to the ESB and its singularity could be the reason why only one BES is fully active. As ΔDOT1B double-expressers have two active BESs, we asked whether a second ESB was present in these cells. To count ESBs, we epitope-tagged RPB6z, a well-characterized subunit of the RNA polymerase I complex that localizes in the nucleolus and the ESB [23]. An N-terminally hemagglutinin (HA)-tagged RPB6z has been shown to be fully functional in the T. brucei procyclic form and to not interfere with Pol I activity in vitro [24]. HA-RPB6z-tagged cell lines were generated in parental and ΔDOT1B cells. PCR and western blotting were used to confirm correct genomic integration and expression levels (unpublished data). ΔDOT1B double-expressers were subsequently selected with G418 and characterized by FACS (unpublished data). We chose ΔDOT1B double-expressing clones in which at least 40% of the cells showed 40–50% of maximum VSG13 expression. IFA with an anti-HA antibody revealed one nucleolus in practically all nondividing cells and at least one extranucleolar spot in 20–46% of both wild-type and ΔDOT1B cells (Figure 6). We detected no statistically significant difference in the number of cells containing one extranucleolar body between parental and ΔDOT1B double-expressers. Approximately 10% of ΔDOT1B double-expressers displayed two small extranucleolar bodies. Surprisingly, the same percentage was found in parental cells, eliminating the possibility that the two extranucleolar spots reflect the location of the two active BES of double-expressers. The nature of a second extranucleolar site remains unclear: it could reflect the replication of the ESB or the presence of an occasional rDNA transcription site outside the nucleolus. In summary, we observed no extra RPB6z-containing site in ΔDOT1B double-expressers. Labeling Pol I transcripts with bromo-UTP also failed to reveal an additional ESB (Figure S2). These results suggest that the partially active BES17 is located in an existing RNA Pol I site, such as the nucleolus or the ESB.

## Discussion

Monoallelic transcription of a single member of a multigene family is a phenomenon seen in several eukaryotes. In mammals, for example, a single olfactory receptor gene is selected from a family of ∼1,000 [25]. For several pathogenic protozoa, this mechanism is the basis for antigenic variation (reviewed in [26]). For example, the human malaria parasite Plasmodium falciparum transcribes only one of ∼60 *var* genes on the surface of infected red blood cells [27]. To switch from one gene to another, most organisms use epigenetic transcriptional control mechanisms that are poorly characterized. In this study, we provide the first evidence that a well-characterized histone methyltransferase, DOT1B, affects antigenic variation in T. brucei on three levels: maintenance of complete BES silencing, rapid transcriptional switching between BESs, and, as a consequence, *VSG* monoallelic expression.

We previously reported that RT-PCR detected no VSG derepression in ΔDOT1B cells [20]. Here, we used a more sensitive approach, quantitative RT-PCR, to quantify the transcript levels of silent *VSG*s. In the absence of DOT1B, silent BESs were 10-fold derepressed in single- and double-expressers, indicating that DOT1B is required for maximum repression of silent BESs. Importantly, disruption of DOT1B does not seem to affect the expression of all genes: no changes were detected in the transcript levels of non-BES genes β*-tubulin* or *actin*, which are transcribed by RNA polymerase II, or *PAG3*, whose transcription by RNA polymerase I is partially repressed at this stage of the life cycle [22]. This selectivity is consistent with what has been described in Saccharomyces cerevisiae, in which loss of Dot1 only derepresses telomeric marker genes and—to a lesser extent—the HM loci [28]. In yeast, this derepression is most likely an indirect consequence of the redistribution of Sir proteins throughout the genome [2]. Although telomeric silencing has been described in T. brucei [29], no causal proteins have been identified. Nevertheless, it is tempting to speculate that disruption of DOT1B causes a similar cascade of events in T. brucei, which results in an indirect derepression of telomeric BESs but not of other loci.

Although silent BESs are partially derepressed in ΔDOT1B cells, the number of G418-resistant clones arising is similar to that of wild-type cells, indicating that partial derepression of a silent BES does not affect the BES switching frequency. This is consistent with previous observations showing that the rate-limiting step of a BES switch is silencing of the previously active BES rather than activation of a new one [30,31]. After 1 wk of selection, all parental G418-resistant clones were puromycin-sensitive and exclusively expressed VSG13, indicating that BES1 was completely and rapidly silenced and BES17 was fully activated. In contrast, after the same time, most ΔDOT1B G418-resistant clones were resistant to both puromycin and G418 and expressed maximum levels of VSG221 and variable levels of VSG13, indicating that two BESs were simultaneously transcribed. To our knowledge, so far, this is the only gene whose disruption leads to the loss of monoallelic *VSG* expression.

A previous study showed, by using a double-BES-tagged cell line similar to ours, that double-resistant clones could be obtained in which ∼65% of the cells simultaneously expressed two VSGs at the surface [12]. There are two important features that distinguish these cells from ΔDOT1B double-resistant clones. First, ΔDOT1B double-resistant clones are obtained by selecting for resistance to only one antibiotic (G418), whereas double-resistant wild-type clones could only be obtained by simultaneously selecting with two antibiotics. Second, ΔDOT1B double-resistant cells are stable and inheritable for at least 30 d in the absence of drug selection, whereas wild-type double-resistant clones rapidly lose double resistance and revert to a 50:50 mixture of trypanosomes with only one of the two marked BESs active. We propose, however, that both studies isolated a comparable switching intermediate, but whereas this is a transient state in wild-type cells, it is stable and inheritable in the absence of DOT1B.

On the basis of FACS and quantitative RT-PCR, we were able to quantify the activities of BES1 and BES17 in switching intermediates. Whereas BES1 remained 100% active, BES17 showed variable expression levels. Interestingly, we never found ΔDOT1B double-expressers that expressed wild-type levels of VSG13, indicating that, although the entire BES17 is transcribed, the level of transcription is not maximal. These results suggest an important *VSG* regulation mechanism that strictly prevents two BESs from being fully transcribed but that is permissive to one BES being fully active and another BES being partially active. We do not know how such a limitation is imposed, but it is possible that it relies on the competition for a limiting transcription factor present in the ESB [10]. This nuclear compartmentalization hypothesis is consistent with our RNA polymerase I localization studies. We observed no difference in the number of RNA Pol I sites between wild-type and ΔDOT1B cells, suggesting that there is a spatial restriction of Pol I transcription sites in the nucleus. We speculate that BES17 is expressed either in the nucleolus or in the unique ESB, close to BES1. This model is consistent with what was observed in double-resistant wild-type clones, in which the two rapidly switching active BESs often were found in close proximity in the nucleus [12], and with the localization of a partially active BES at the periphery of the nucleolus [32]. Despite the localization in a Pol I competent compartment, BES17 is only partially activated, which suggests that another limiting factor is missing. We cannot exclude the possibility that the levels of expression of BES17 are too low to allow the detection of an additional RPB6z site, but this seems unlikely because, for these experiments, we chose ΔDOT1B double-expressers where at least 40% of the cells were displaying 40–50% of maximal VSG13 expression. If BES17 is transcribed by half the amount of Pol I complexes than BES1, then we should still be able to detect a bright spot.

After 8–24 d of continuous G418 selection, switching intermediates eventually express only VSG13, suggesting that the competition for a limiting factor is resolved in some double-resistant cells. Consequently, this leads to the rapid silencing of BES1 and full activation of BES17. BES switching is most likely a complex mechanism that involves several events in a precise order. Nothing is known about the intermediates of a BES switch, which means that we cannot exclude the possibility that double-resistant cells are not a switching intermediate but simply a fraction of the population selected by drug pressure. If the latter was true, then one would predict that the number of G418-resistant clones would be higher in ΔDOT1B because the drug would select not only for VSG13 expressers but also for the fraction of cells with higher transcription levels in BES17. Also, if drug double-expressers were a product of drug selection, then, when pressure is removed, one would predict that the double-resistance phenotype should be lost, as demonstrated by [12] and discussed above. None of these predictions is consistent with our data, suggesting that double-expressers are more likely switching intermediates and, consequently, DOT1B is involved in the kinetics of switching.

The VSG profile detected by FACS indicates that ΔDOT1B double-expressers have the same amount of VSG221 molecules at the surface as a VSG221 expresser and an extra 5–90% of the total VSG13 usually present in a VSG13 expresser. As VSG molecules form a tightly packed surface coat, we speculate that the volume of the cells might be slightly larger to accommodate the excess of VSG molecules. A similar observation was made previously, when two VSGs were simultaneously expressed at the same levels as one normally is [33]. By means that we can only speculate upon, the cells seem able to accommodate up to twice as much VSG expression.

Several studies suggest that silencing of a previously active BES and activation of a new BES are tightly coupled (reviewed in [34]), which predicts the existence of a sensing mechanism that facilitates cross-talk between BESs. It remains unclear what step of switching is affected in ΔDOT1B: activation of BES17 or silencing of BES1. If DOT1B is a transcription activator, as shown in cancer cells [7], then its absence may interfere with BES17 activation. In this scenario, double-resistant cells are a consequence of a defective BES17 activation that leads, due to incomplete cross-talk between BESs, to BES1 remaining fully active. Alternatively, the effects of DOT1B depletion may be an indirect consequence of a disruption of telomeric silencing. In this case, the absence of DOT1B may interfere with silencing of BES1, and by cross-talk, a second BES is temporarily prevented from being fully activated. Interestingly, no population of ΔDOT1B double-expressers ever was found with intermediate levels of VSG221, indicating that, even in the absence of DOT1B, BES1 either remains fully active or becomes fully silenced. These results indicate that DOT1B is not implicated in the all-or-nothing property of the machinery that silences a previously active BES.

BES switching probably requires the ordered recruitment of complex machinery that recognizes a specific chromatin configuration and remodels it to establish a new chromatin structure in the two involved BESs. In this study, we showed that a histone-modifying enzyme is part of this machinery, which unveils the importance of chromatin modifications in antigenic variation of African trypanosomes.

## Materials and Methods

### Strains and media.

T. brucei bloodstream-form parasites (strain Lister 427, antigenic type MiTat 1.2, clone 221a) [35] were cultured at 37 °C in HMI-9 medium [36]. The “single marker” cell line expresses T7 RNA polymerase and the tet repressor, allowing inducible expression of ectopic genes under control of the T7 promoter and tet operator [37]. Stable transfections were performed as described using either a BTX electroporator [38] or an AMAXA Biosystems nucleofector. In the latter, 40 million cells were resupended in T-cell solution and transfected using program X-001. DOT1B alleles (Accession Number: XM_001218759) were deleted using a PCR-based approach that conferred resistance to hygromycin and phleomycin as described previously [39]. Loss of both alleles was confirmed by PCR and western blotting with an anti-H3K79me3 antibody [20]. In drug sensitivity assays, 10^4^ cells were diluted in 10 ml of HMI-9 containing either 100 μg/ml of G418 or 1 μg/ml of puromycin. Cell growth was determined 48 h later. To measure population doubling time, cell growth was monitored for at least 8 d under similar conditions.

### Generation of a switching-reporter strain and tagged proteins.

An in situ switching reporter strain was derived from wild-type clone 221a, in which *PUR* and *NEO* were integrated downstream of different BES promoters. Integration of PUR in BES1 was obtained by transfecting 221a cells with a construct (pLF12) consisting of three fragments cloned into pBluescriptSKII+ in the following 5' to 3' order: the upstream promoter region (UP), *PUR* flanked by the Aldolase splice acceptor site and 3' UTR, and the downstream promoter region (DP) (Figure S3). UP is the 1590-bp fragment spanning from the 50-bp repeat array to 240 bp downstream of the ES transcription start site. DP is the 1150-bp region spanning from 287 bp downstream of the ES transcription start site to *ESAG7*. The BES1/PUR cell line was subsequently transfected with pLF13, which introduced *NEO* in an identical position of a second random BES. After multiple clones were genotyped, the cell line chosen as in situ switch reporter integrated PUR at the actively transcribed BES1 and NEO at the silent BES17. Because it expresses VSG221, this cell line was named PN221.

An influenza HA tag was introduced at the 5' end of one of the RPB6z alleles by transfection with pLF96, derived from a pPURO-HA-RPB6z construct [24] in which the PURO cassette was replaced by a HindIII to SacI fragment encoding blasticidin resistance, flanked upstream by the hsp70 intergenic region and downstream by the β-tubulin intergenic region. HA-RPB6z-tagged cell lines were generated in single marker and ΔDOT1B backgrounds. Single marker was preferred over wild-type 221 for future uses of the tagged cell line.

### Real-time quantitative RT-PCR and northern blot analysis.

Total RNA was extracted from ∼10^8^ bloodstream-form cells with RNA STAT-60 (TEL-TEST, Inc.), following the manufacturer's instructions. Approximately 20 μg of RNA was DNAse-treated, phenol–chloroform-extracted, and ethanol-precipitated. cDNA was synthesized with oligodT(18) primers using the StrataScript First Strand Synthesis System (Stratagene). Approximately 5 μl of three serial dilutions of cDNA (1:10, 1:40, and 1:160) was used for quantitative RT-PCR with SYBR green (Applied Biosystems). Amplification reactions were performed in duplicates in an Applied Biosystems 7900HT. Primer sequences are listed in Table S1. Relative levels of gene expression were measured using the comparative cycle threshold C_T_ method (User Bulletin 2, ABI, http://www.appliedbiosystems.com/), using β-tubulin or actin as reference genes. For northern analysis, 3 μg of total RNA was resolved on a 1.5% formaldehyde agarose gel, transferred to a nylon membrane, and detected with a random primer-labelled specific probe. Sequences are available upon request.

### Anti-VSG antibodies.

Anti-VSG13 (also known as MITat1.13) serum was obtained by immunizing a rabbit with purified native VSG13, as described previously [40]. Rabbit anti-VSG13 and rabbit and chicken anti-VSG221 (MITat1.2) were depleted of glycosylphosphatidylinositol-related VSG cross-reacting-determinant (CRD) antibodies by passing through an affinity column of a different VSG [41]. For FACS, live cells were stained with affinity-purified antibodies directly conjugated to Alexa Fluor 488 (anti-VSG221) or Alexa Fluor 647 (anti-VSG13).

### VSG immunodetection.

VSG expression was assayed by dot blotting. Briefly, 2–3 × 10^6^ trypanosomes were concentrated into 10 μl of HMI-9 medium by centrifugation at 2,500*g* for 4 min. Approximately 2 μl of cell suspension was dropped onto a Hybond-ECL membrane (Amersham), previously incubated in PBS pH 7.2 for 5 min. After being air-dried for 5 min, the membrane was processed as for western blotting. Primary anti-VSG antibodies were detected with horseradish-peroxidase-conjugated sheep anti-rabbit antibodies (Amersham).

### Immunofluorescence.

For VSG visualization, cells were fixed in 2% (w/v) formaldehyde at room temperature for 10 min. After being washed twice in PBS containing 2 mM EDTA, cells were settled onto aminopropyltriethoxysilane-coated coverslips. Immobilized cells were blocked in PBG (PBS containing 0.2% cold fish gelatin (Sigma) and 0.5% BSA) and incubated with chicken ΔCRD anti-VSG221 and rabbit ΔCRD anti-VSG13 sera for 1 h followed by tetramethylrhodamine isothiocyanate-conjugated donkey anti-rabbit IgG (Jackson ImmunoResearch Laboratories) or Alexa Fluor 488-conjugated goat anti-chicken IgG (Invitrogen) for 1 h. Cells were mounted in DAPI-containing antifade medium Vectashield (Vector Laboratories) and analyzed with a Nikon Optiphot microscope. The same protocol was followed for HA-RPB6z detection, except cells were fixed in 0.5% (w/v) formaldehyde for 5 min. After settling onto coverslips, cells were permeabilized with 0.2% NP40 for 5 min. The HA tag was detected with a monoclonal anti-HA.11 antibody, clone 16B12 (Covance). Coverslips were examined using DeltaVision deconvolution microscopy (Applied Precision).

### FACS analysis and cell sorting.

For quantification of cell-surface VSG, 10^6^ cells were centrifuged in a previously chilled microtube at 2,500*g* for 4 min at 4 °C. Cells were resuspended in 100 μl of cold HMI-9 to which Alexa Fluor 488 anti-VSG221 and/or Alexa Fluor 647 anti-VSG13 conjugated antibodies had been added. After 20 min of incubation at 4 °C with gentle shaking, cells were washed three times in cold HMI-9, resuspended in cold HMI-9, and immediately analyzed on a FACSort flow cytometer (Becton Dickinson Biosciences). Data were processed with FlowJo software (Tree Star). Approximately 4 × 10^6^ cells were similarly stained for sorting on a FACSAria cell-sorter (Becton Dickinson Biosciences). Sorted cells were immediately washed twice in HMI-9 containing 400 U/ml penicillin and 400 μg/ml streptomycin. Approximately 5, 100, or 2,000 cells/ml were transferred to 24-well plates without or with 100 μg/ml G418. The VSG profiles of subclones were analyzed after 6–8 d.

## Supporting Information

Figure S1 Growth of a ΔDOT1B Class II Clone under Different Drug Regimes (1.1 MB AI)

Figure S2 Frequency of Pol I Transcript Foci Is Identical in Parental and ΔDOT1B Double-Expresser Clones (A) Examples of four nondividing bloodstream cells in which transcripts were labeled with bromo-UTP and visualized by IFA. α-Amanitin inhibits transcription by RNA Pol II and III, allowing the exclusive detection of RNA polymerase I transcripts. DNA was detected with DAPI (blue). Scale bar: 2 μm.
(B) Quantification of Pol I transcript foci in parental and ΔDOT1B double-expresser clones, in different stages of the cell cycle. An average of 40 cells/clone was counted.
(1.5 MB PDF)

Figure S3 Constructs to Generate Reporter Strain pLF12 and pLF13 constructs used to introduce drug-selectable markers into BESs. Dashed lines represent the targeting sequences UP and DP. Gray boxes represent the aldolase splice-acceptor site and 3' UTR. pLF12 and pLF13 differ only in the drug-selectable marker. Genotyping of clones obtained after transfection of both plasmids into wild-type 221 cells led to the identification of the PN221 cell line in which pLF12 had targeted the actively transcribed BES1 and pLF13 the silent BES17.
(1.1 MB AI)

Table S1 Primer Sequences Used for Quantitative RT-PCR (1.1 MB AI)

## Abbreviations

BES: bloodstream expression site
CRD: cross-reacting-determinant
DOT: disruptor-of-telomeric silencing
DP: downstream promoter region
ESB: expression site body
FACS: fluorescent-activated cell sorting
HA: hemagglutinin
IFA: immunofluorescence analysis
*NEO*: neomycin resistance gene
*PAG3*: procyclin-associated gene 3
*PUR*: puromycin resistance gene
RT-PCR: reverse-transcriptase PCR
UP: upstream promoter region
VSG: variant surface glycoprotein
