# Supplementary material for: A Histone Methyltransferase Modulates Antigenic Variation in African Trypanosomes
Source: PLoS Biol. 2008 Jul 1;6(7):e161. doi: 10.1371/journal.pbio.0060161 (PMC2443197; doi:10.1371/journal.pbio.0060161)
Supplement: Supplementary file 1 [file pbio.0060161.sg001.pdf]

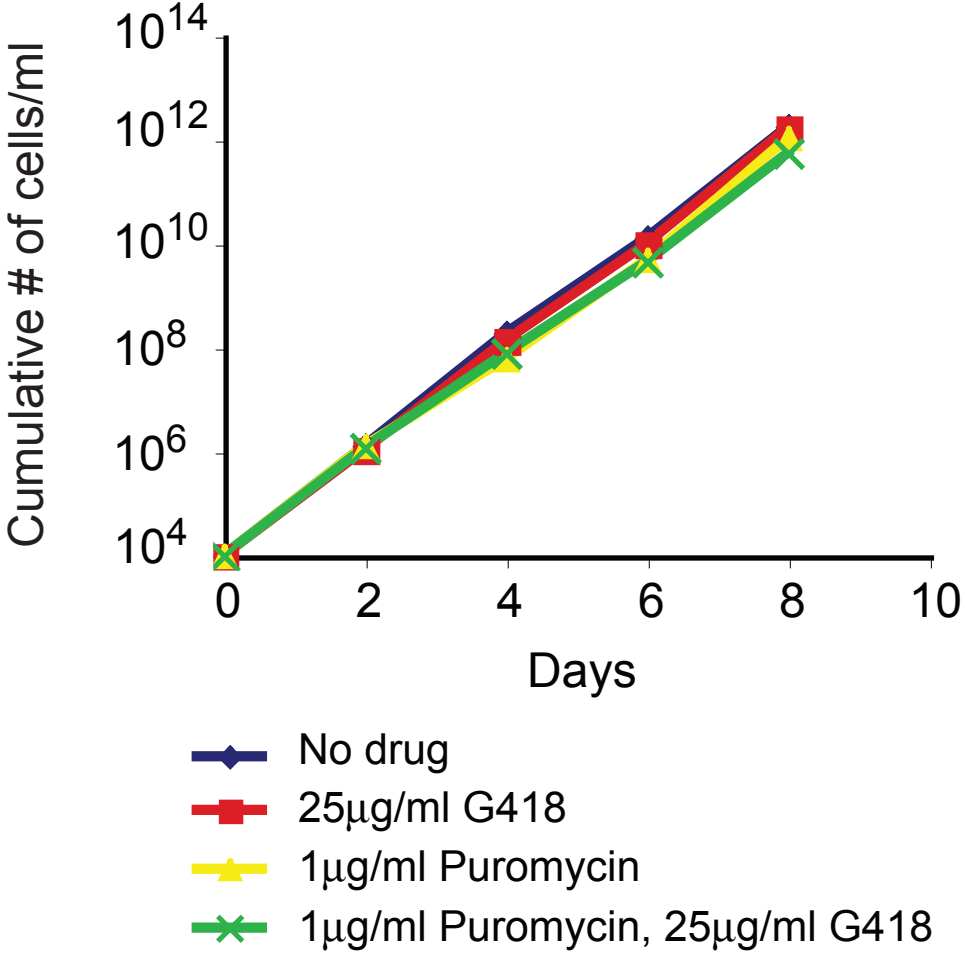

Cumulative # of cells/ml
10^14
10^12
10^10
10^8
10^6
10^4
0
2
4
6
8
10
Days
No drug
25µg/ml G418
1µg/ml Puromycin
1µg/ml Puromycin, 25µg/ml G418
