# Supplementary material for: A Histone Methyltransferase Modulates Antigenic Variation in African Trypanosomes
Source: PLoS Biol. 2008 Jul 1;6(7):e161. doi: 10.1371/journal.pbio.0060161 (PMC2443197; doi:10.1371/journal.pbio.0060161)
Supplement: Supplementary file 2 [file pbio.0060161.sg002.pdf]

Figure S2

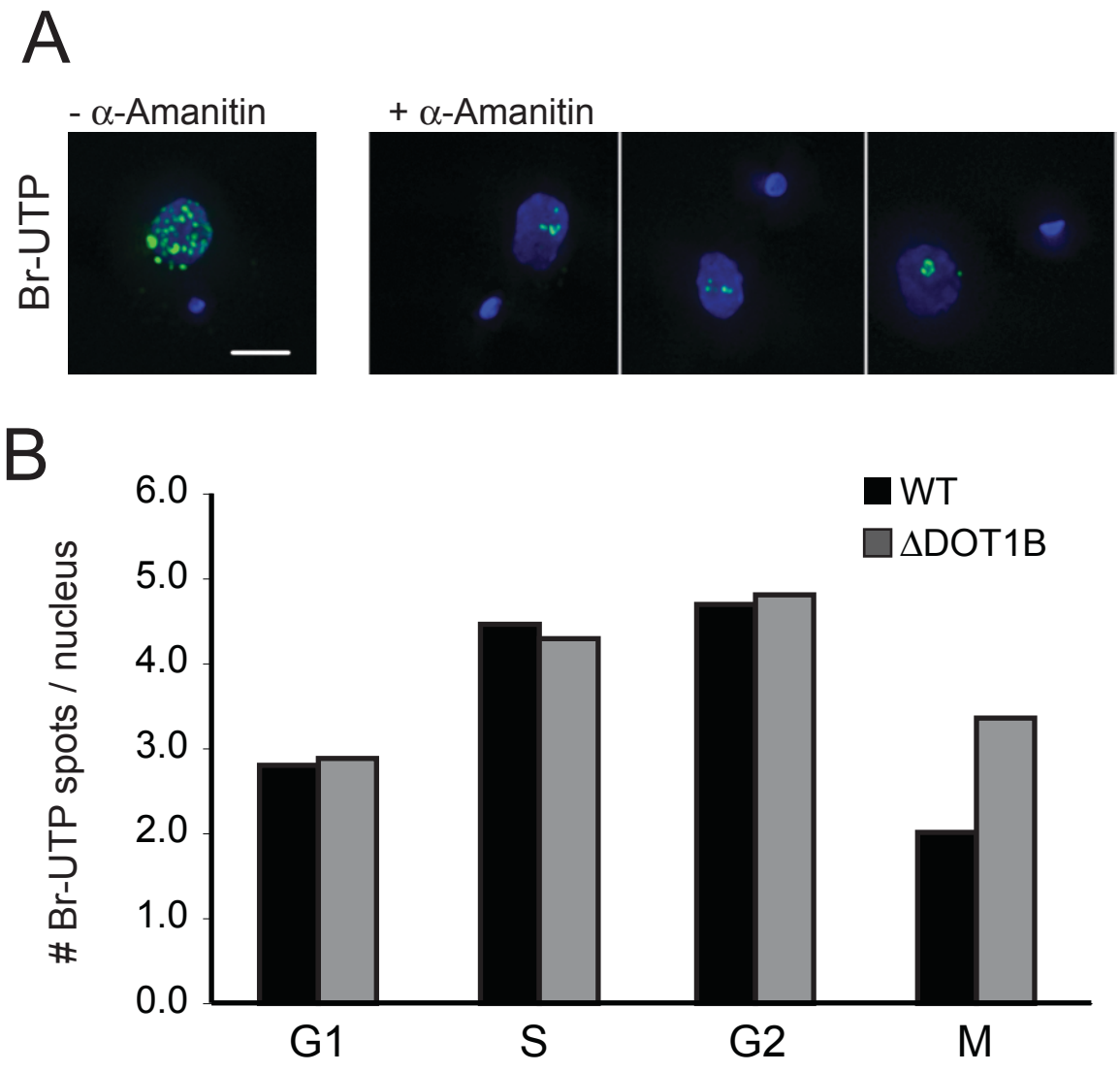

A
- α-Amanitin
+ α-Amanitin
Br-UTP
B
Br-UTP spots / nucleus
6.0
5.0
4.0
3.0
2.0
1.0
0.0
G1
S
G2
M
WT
ΔDOT1B
