# Supplementary material for: A Histone Methyltransferase Modulates Antigenic Variation in African Trypanosomes
Source: PLoS Biol. 2008 Jul 1;6(7):e161. doi: 10.1371/journal.pbio.0060161 (PMC2443197; doi:10.1371/journal.pbio.0060161)
Supplement: Supplementary file 3 [file pbio.0060161.sg003.pdf]

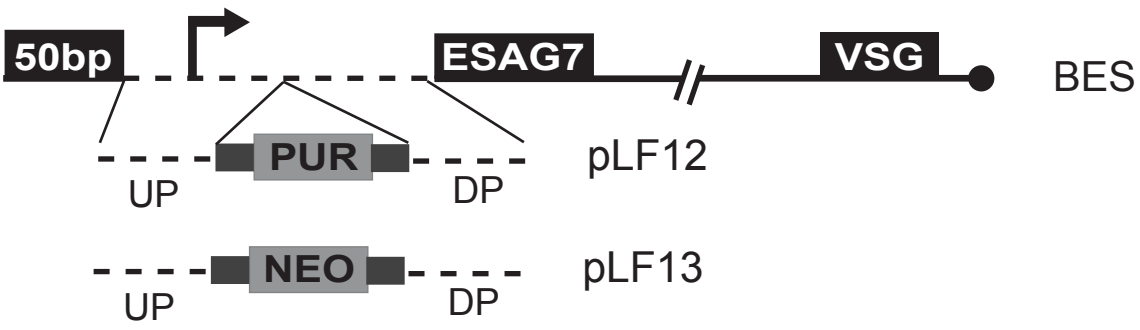

50bp
ESAG7
VSG
BES
PUR
pLF12
UP
DP
NEO
pLF13
UP
DP
