# Supplementary material for: A Histone Methyltransferase Modulates Antigenic Variation in African Trypanosomes
Source: PLoS Biol. 2008 Jul 1;6(7):e161. doi: 10.1371/journal.pbio.0060161 (PMC2443197; doi:10.1371/journal.pbio.0060161)
Supplement: Supplementary file 4 [file pbio.0060161.st001.pdf]

Table S1

Gene	Forward oligo		Reverse oligo	
	Name	Sequence	Name	Sequence
PAG3	155	GCAAATGCTCCTCTTCTCC	156	TGGCTGCCGAAGCAAGCG
Actin	150	CGGACGAGGAACAAACTGC	151	TTTCCATGTCATCCCAATTGG
β-Tubulin	142	TTCAGGCTGGCCAATGCG	143	TACGGAGTCCATTGTACCTG
VSG 221 / MITat1.2	74	AGCAGCCAAGAGGTAACAGC	82	CAACTGCAGCTTGCAAGGAA
VSG 13 / MiTat1.13	78	ATAACGCATGGCCATCTTGAC	79	GTCGTTGCTGTGGATTGCTC
VSG 224 / MiTat1.3	52	GACGCAGCAGAATCAACAC	45	GCTTATTTTGTGTCTGTCGC
VSG VO2 / MiTat1.9	51	ACTAAGCTCGTGGCGCAC	141	CGCGTAGTTGACGCATGAC
VSG 800 / MiTat1.18	117	ACAGACCGCCGACAGTATC	140	GTATCTTTGTAGGCCGCTGC
NEO	77	CTTGCCGAATATCATGGTGGA	83	ACCGTAAAGCACGAGGAAGC
